# Estimated Effect of Inactivated Poliovirus Vaccine Campaigns, Nigeria and Pakistan, January 2014–April 2016

**DOI:** 10.3201/eid2302.161210

**Published:** 2017-02

**Authors:** George Shirreff, Mufti Zubair Wadood, Rui Gama Vaz, Roland W. Sutter, Nicholas C. Grassly

**Affiliations:** Imperial College London, London, UK (G. Shirreff, N.C. Grassly);; World Health Organization (WHO), Islamabad, Pakistan (M.Z. Wadood);; WHO, Abuja, Nigeria (R.G. Vaz);; WHO Global Polio Eradication Initiative, Geneva, Switzerland (R.W. Sutter)

**Keywords:** poliovirus, polio, vaccines, viruses, epidemiology, Nigeria, Pakistan, inactivated poliovirus vaccine, IPV, vaccine campaign, mass campaign, vaccination, immunization, trivalent oral polio vaccine

## Abstract

In 2014, inactivated poliovirus vaccine (IPV) campaigns were implemented in Nigeria and Pakistan after clinical trials showed that IPV boosts intestinal immunity in children previously given oral poliovirus vaccine (OPV). We estimated the effect of these campaigns by using surveillance data collected during January 2014–April 2016. In Nigeria, campaigns with IPV and trivalent OPV (tOPV) substantially reduced the incidence of poliomyelitis caused by circulating serotype-2 vaccine–derived poliovirus (incidence rate ratio [IRR] 0.17 for 90 days after vs. 90 days before campaigns, 95% CI 0.04–0.78) and the prevalence of virus in environmental samples (prevalence ratio [PR] 0.16, 95% CI 0.02–1.33). Campaigns with tOPV alone resulted in similar reductions (IRR 0.59, 95% CI 0.18–1.97; PR 0.45, 95% CI 0.21–0.95). In Pakistan, the effect of IPV+tOPV campaigns on wild-type poliovirus was not significant. Results suggest that administration of IPV alongside OPV can decrease poliovirus transmission if high vaccine coverage is achieved.

The live attenuated oral poliovirus vaccine (OPV) is cheap and easy to administer; therefore, it has been the vaccine of choice for the Global Polio Eradication Initiative. However, the immunogenicity and efficacy of OPV is reduced in tropical developing countries, perhaps as a result of the high burden of other enteric pathogens (*1*,*2*). Furthermore, although OPV offers lifelong protection against paralytic poliomyelitis, intestinal immunity against infection and poliovirus shedding appears to wane quite rapidly (*3*), meaning that OPV-immunized persons may contribute to community transmission of poliovirus (*4*,*5*). OPV also causes vaccine-associated paralytic poliomyelitis in ≈1 child per million vaccinated, and, in rare instances, may revert to a neurovirulent and transmissible form, causing outbreaks of poliomyelitis associated with vaccine-derived polioviruses (VDPV) (*6*,*7*). 

In developing countries, seroconversion induced by inactivated poliovirus vaccine (IPV) is better than that induced by trivalent OPV (tOPV); thus, in these countries, IPV provides better protection against paralytic poliomyelitis (*8*,*9*). However, immunization with IPV alone induces very limited intestinal mucosal immunity against viral shedding compared with that induced by OPV, and therefore, IPV may be permissive to poliovirus circulation even when vaccination coverage is high (*10*,*11*). Recent studies showed that in children previously exposed to OPV (and therefore mucosally primed), the administration of 1 dose of IPV substantially boosted intestinal immunity against fecal shedding of poliovirus measured after challenge with the live attenuated OPV (*12*,*13*). Of note, this boost was greater than that observed after an additional dose of OPV.

These encouraging findings motivated the introduction, beginning in 2014, of IPV to mass vaccination campaigns in Nigeria and Pakistan, 2 countries that had circulating VDPV and wild-type poliovirus at that time. However, it is not yet known whether the IPV boost of intestinal immunity observed against a challenge dose of vaccine (Sabin) poliovirus will translate to an effect of IPV campaigns on transmission of wild-type polioviruses and VDPVs at the community level.

To determine the effectiveness of campaigns using IPV+tOPV or tOPV alone in preventing the circulation of wild-type polioviruses or VDPVs, we analyzed poliovirus surveillance data for 2014–2016 from Nigeria and Pakistan. We report the results of that analysis and discuss their implications for the polio endgame strategy.

## Materials and Methods

### Data

We analyzed polio vaccination data reported from Nigeria and Pakistan during January 1, 2014–April 30, 2016, by combining information on the dates and locations (districts) of vaccination campaigns, the district-level incidence of poliomyelitis reported through surveillance for acute flaccid paralysis, and the presence or absence of poliovirus in environmental samples collected at regular intervals (weekly, every 2 weeks, or monthly) from wastewater/sewage channels that flow out of areas of interest for environmental surveillance (*14*). We obtained the data on July 11, 2016, from PolIS (the Polio Information System), which collects poliovirus-associated data and information, including surveillance data, from different sources for the World Health Organization (WHO) and its partners. (Acute flaccid paralysis and supplemental immunization activity data are available from the WHO Institutional Data Access/Ethics Committee for researchers who meet the criteria for access to confidential data.) We estimated the number of children 0–14 years of age living in each district in Nigeria and Pakistan by using WorldPop (http://www.worldpop.org.uk) gridded population maps for 2014 (Nigeria) and 2015 (Pakistan) with administrative boundary data from WHO and country-specific age-distribution estimates from the United Nations Population Division (*15*).

### Statistical Analysis

We defined the incidence rate ratio (IRR) for a campaign as the rate of reported poliomyelitis cases in the period after the campaign divided by the rate reported before the campaign. We focused on a 90-day period before and after the campaign, but we explored the sensitivity of our results to the choice of the length of this time period. We created a line-list containing every campaign for each district in Pakistan and Nigeria and recorded the 1) campaign location (district and state/province); 2) vaccine(s) used (i.e., IPV+tOPV, tOPV alone, bivalent OPV [bOPV] alone, or serotype-1 monovalent OPV [mOPV1] alone); 3) polio incidence before and after the campaign; and 4) length of the observation period. To avoid including information more than once in the statistical analysis, we censored data following entry into the line-listed database. To avoid bias, we entered campaigns with each vaccine(s) into the database in random order, according to a sequence of vaccine types (i.e., IPV+tOPV, tOPV, bOPV, mOPV); this system of entry maximized available information on campaigns that used IPV+tOPV and their effect compared with campaigns that used tOPV alone.

We used a Poisson regression model to estimate the IRR by vaccine type(s) administered in a campaign. The incidence of poliomyelitis in a district was modeled as a log-linear function of the following 3 independent variables: 1) an indicator variable (i) for whether observations were before or after a campaign; 2) the natural log of the number of child-years of observation (t) with a coefficient equal to 1, such that the incidence rate per child-year was modeled; and 3) an interaction term, such that the change in incidence after a campaign depended on a categorical variable (v) describing the type of vaccine(s) used in the campaign. We used a mixed-effects model with a random effect for each state or province and vaccine combination on the model intercept (baseline incidence). We fit this model using the lme4 package in the R statistical programming language [regression equation: n_cases ~ i:v + (1|state:v), offset = log(t)], where : indicates an interaction term and (1|state:v) indicates the random effect on the intercept) (*16*,*17*). We compared this mixed-effects regression model with a model that included only the fixed effects using the Akaike Information Criterion (AIC). We used a similar approach with a mixed-effects binomial regression model to estimate the prevalence ratio (PR) for the proportion of environmental samples that were positive for poliovirus isolation before and after campaigns with different vaccines.

## Results

### Nigeria

**Figure 1:**
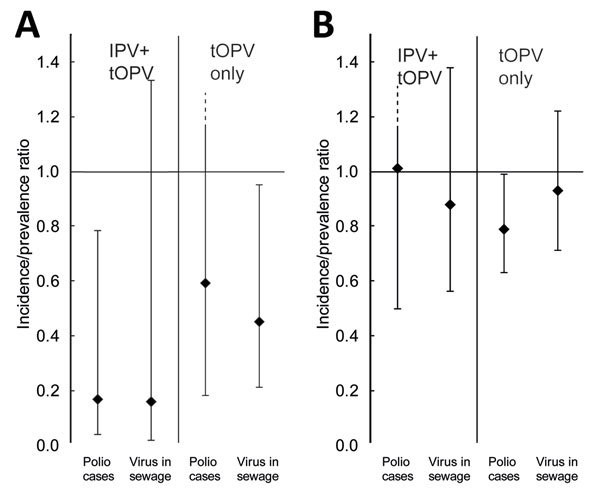
Effect of mass vaccination campaigns with inactivated poliovirus vaccine plus trivalent oral poliovirus vaccine (IPV+tOPV) or tOPV alone on poliovirus detection in persons or the environment, Nigeria and Pakistan, 2014–2016. The incidence rate ratio for poliomyelitis and the prevalence ratio for poliovirus detection in environmental samples (sewage) during 90 days after compared with 90 days before mass vaccination campaigns are shown for Nigeria (A) and Pakistan (B) and can be compared with the complete data and estimates (Technical Appendix Tables 1, 3). The estimates (diamonds) are shown with 95% CIs (error bars); the dashed error bars indicate when the upper CI exceeded the plot limit of 1.4.

In Nigeria, 5 campaigns with IPV+tOPV in different locations and at different scales took place during the analysis period, resulting in 55 district-campaign observations (Technical Appendix Table 1). These campaigns used a total of 3.8 million doses of IPV and targeted children 14 weeks–59 months of age; although, 1 campaign included only children 6–35 months of age. The campaigns were followed by a reduction in the incidence of poliomyelitis associated with circulating VDPV serotype 2 (cVDPV2). The IRR, based on the mixed-effects regression, for cases in the 90 days after the campaigns compared with 90 days before the campaign was 0.17 (95% CI 0.04–0.78; p = 0.023) (Figure 1; Technical Appendix Table 1). After these campaigns, the prevalence of cVDPV2 was reduced among environmental samples collected in the same districts as the campaigns; however, this reduction was not statistically significant (PR 0.16 based on the mixed-effects regression, 95% CI 0.02–1.33; p = 0.09). Campaigns with tOPV alone did not significantly reduce the incidence of poliomyelitis associated with cVDPV2 (IRR 0.59, 95% CI 0.18–1.97; p = 0.215 for comparison with IPV+tOPV campaigns), but they did significantly reduce the prevalence of cVDPV2 in the environment (PR 0.45, 95% CI 0.21–0.95; p = 0.369 for comparison with IPV+tOPV campaigns). As expected, campaigns with bOPV containing serotypes 1 and 3 did not show any effect on cVDPV2 prevalence or poliomyelitis cases. In areas of Nigeria where IPV campaigns were conducted, the number of polio cases associated with wild-type poliovirus (n = 1) was insufficient to enable a comparable analysis for this poliovirus type. Changing the length of time examined before and after each campaign did not substantially change the results. Reducing the period to <90 days led to a loss of statistical power; increasing the period to 150 days, resulted in a statistically significant effect of IPV+tOPV campaigns on the prevalence of cVDPV2 in environmental samples (Technical Appendix Table 2).

**Figure 2:**
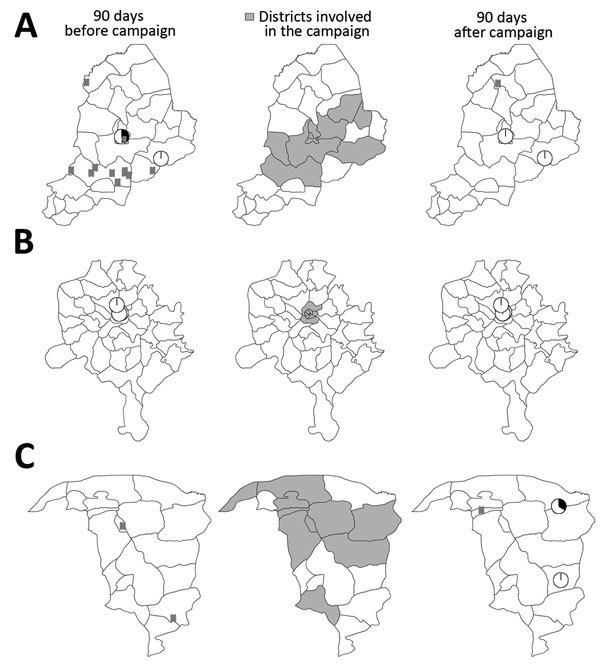
Data used to estimate the effect of inactivated poliovirus vaccine (IPV) campaigns in Nigeria. Maps show location of poliomyelitis cases associated with circulating serotype 2 vaccine–derived poliovirus (cVDPV2) and prevalence of this virus in the environment 90 days before and after campaigns with IPV plus trivalent oral poliovirus vaccine (IPV+tOPV) in Borno during June 2014 (A), Kano during March 2015 (B), and Yobe during November 2014 (C). Locations of cVDPV2 cases (rectangles) and environmental surveillance sites (pie charts) are plotted by district; locations within districts are plotted randomly. Pie charts are colored according to the proportion of environmental samples positive (black) or negative (white) for cVDPV2 during the 90-day period. cVDPV2, circulating serotype 2 vaccine-derived poliovirus IPV, inactivated poliovirus vaccine; tOPV, trivalent oral poliovirus vaccine. Publication of these maps does not imply the expression of any opinion whatsoever on the part of the World Health Organization concerning the legal status of any territory, city, or area or of its authorities or concerning the delimitation of its frontiers or boundaries.

The mixed-effects regression models gave a significantly better fit to the data compared with models that included only fixed effects, indicating heterogeneity among states in the incidence of poliomyelitis and prevalence of poliovirus isolation; the AIC for mixed versus fixed-effects models was 110.4 versus 194.9 for the IRR and 137.5 versus 171.6 for the PR. This heterogeneity is also apparent in plots of the location of poliomyelitis cases and environmental surveillance results before and after campaigns with IPV+tOPV (Figure 2).

### Pakistan

During the analysis period in Pakistan, 15 IPV+tOPV campaigns were conducted, resulting in a total of 133 district-campaign observations (Technical Appendix Table 3). These campaigns targeted children up to 23 months of age and used a total of 3.9 million doses of IPV. The effect of these campaigns on the number of cases of poliomyelitis associated with wild-type poliovirus serotype 1 (WPV1) and isolation of this virus in the environment was not apparent (IRR 1.01, 95% CI 0.50–2.02; PR 0.88, 95% CI 0.56–1.38) (Figure 1; Technical Appendix Table 3). Campaigns using only tOPV had a modest but statistically significant effect on the incidence of poliomyelitis associated with WPV1 (IRR 0.79, 95% CI 0.63–0.99; p = 0.039). However, this effect was not apparent from environmental data (PR 0.93, 95% CI 0.71–1.22; p = 0.586). In areas of Pakistan where IPV campaigns were conducted, the number of poliomyelitis cases associated with cVDPV2 (n = 2) was insufficient to enable analysis. Changing the length of time examined before and after each campaign did not substantially change these results, although the effect of campaigns with IPV+tOPV on the incidence of poliomyelitis became significant when the period was increased to 150 days (online Technical Appendix Table 4).

Our results showed evidence for significant heterogeneity in the incidence of poliomyelitis and prevalence of WPV1 isolation among provinces in Pakistan. The AIC for mixed-effects versus fixed-effects models was 187.3 versus 1393.3 for the IRR and 133.7 versus 169.3 for the PR.

## Discussion

We assessed the effect of vaccination campaigns that used IPV alongside OPV on the transmission of wild-type and VDPV. In Nigeria, we observed a substantial reduction in the incidence of poliomyelitis associated with cVDPV2 and in the detection of cVDPV2 in environmental samples after mass campaigns with IPV+tOPV. This reduction was greater than that observed after campaigns that used tOPV alone, although the difference was not statistically significant. This finding suggests that the substantial boost to intestinal immunity after vaccination with IPV, as observed in recent OPV challenge studies (*12*,*13*), translates into a measurable effect of IPV campaigns on poliovirus transmission in the community. These encouraging findings support the use of IPV in campaigns during the polio endgame to eradicate remaining wild-type polioviruses, increase population immunity in advance of withdrawal of OPV serotypes, and respond to any outbreaks of vaccine-derived poliovirus. These results are particularly relevant in the context of OPV withdrawal, which began with the globally synchronized withdrawal of OPV serotype 2 (OPV2) in April 2016, because IPV has the advantage of providing a boost in mucosal immunity while avoiding release of new Sabin viruses that could evolve to VDPVs.

In Pakistan, an effect of mass campaigns with IPV+tOPV on persistent WPV1 circulation was not apparent. This finding may partly reflect a lack of statistical power. In the sensitivity analysis, using a longer time window of 150 days resulted in a significant reduction in the incidence of poliomyelitis: IRR 0.56 (95% CI 0.33–0.95) for IPV+tOPV campaigns and IRR 0.67 (0.53–0.86) for tOPV-only campaigns. However, this reduction was not apparent for WPV1 isolated in the environment (PR 0.85 [95% CI 0.59–1.23] for IPV+tOPV campaigns, and PR 1.00 [95% CI 0.76–1.30] for tOPV-only campaigns). In Pakistan, the lack of evidence for an effect from IPV campaigns may also reflect low coverage during the campaigns and restrictions on the age groups that were targeted for vaccination. Campaign coverage was suboptimal in Pakistan during much of 2014–2015; in September 2015, in areas at high risk for polio, just 39% of union councils were estimated to have achieved >80% campaign coverage, although this improved to 62% in November 2015 (*18*). Estimates of campaign coverage during January 2014–June 2015, which were based on the vaccination histories of children reported with nonpolio acute flaccid paralysis, also suggest that coverage among undervaccinated communities in Pakistan was poorer than that in Nigeria (*19*). Suboptimal vaccination coverage means that fewer children will benefit from a boost in intestinal immunity by IPV, not only as a result of smaller numbers receiving the vaccine, but also because fewer children will have been mucosally primed by OPV given in earlier campaigns or though routine immunization systems. According to WHO/UNICEF, routine immunization coverage with OPV in 2015 was suboptimal (≈75% on average) in both Pakistan and Nigeria.

During vaccination campaigns, it is more challenging to deliver IPV than OPV because IPV must be administered by trained healthcare workers and is thus offered at specific locations only (e.g., health centers) rather than being delivered directly to households, as with OPV. Nonetheless, in Nigeria and Pakistan, IPV campaign coverage has been reported to be comparable to that for OPV-only campaigns (*20*,*21*). However, in Pakistan, IPV was administered only to children <2 years of age, and in Nigeria (with the exception of 1 campaign), children <5 years of age were included. The wider age group in Nigeria may have contributed to the greater effect of IPV on poliovirus transmission because children 2–4 years of age may shed poliovirus and contribute to transmission despite being protected against poliomyelitis.

The difference in the effect of campaigns that used IPV in Pakistan and Nigeria may also reflect differences in the circulating poliovirus types (cVDPV2 in Nigeria and WPV1 in Pakistan). However, this seems unlikely because the transmissibility and pathogenicity of cVDPV2 in Nigeria appears equivalent to that for wild-type poliovirus (*22*).

Our analysis has several limitations, including its observational nature and lack of randomization of vaccines used during campaigns, which could have resulted in systematic differences in the areas that used IPV+tOPV compared with tOPV alone. We attempted to account for these differences in our statistical analysis by allowing for random variation in the incidence of poliomyelitis and prevalence of virus isolation by state or province and by vaccine(s) used in the campaign. The statistical power of our analysis was also limited by the low incidence of poliomyelitis in areas with IPV+tOPV campaigns. In Nigeria, the estimate of the effect of IPV+tOPV campaigns against poliomyelitis was driven by the results from Borno State, where an emerging cVDPV2 outbreak was apparently stopped by these campaigns. Environmental surveillance data from other states (Sokoto, Kaduna) support the effectiveness of IPV in campaigns, but the number of sampling sites informing the estimates was limited. As further experience with the use of IPV in campaigns is acquired, and with the planned expansion of environmental surveillance, it will be possible to refine our estimates. It may also become possible to increase the strength of our analyses by using additional statistical techniques, such as interrupted time-series methods, which are currently not appropriate, given the limited number of observations. Although a cluster-randomized trial would deal with any biases introduced through a lack of randomization in our study, such a trial would not be ethical, and to achieve sufficient statistical power would require such a large study as to be impractical.

Another limitation of our study is that we used poliovirus isolation in environmental (wastewater/sewage) samples as a proxy for poliovirus transmission in the community. Although environmental surveillance is known to be highly sensitive for poliovirus circulation in the catchment population, even in the absence of poliomyelitis cases, it may not capture more subtle effects of vaccination campaigns on the extent of poliovirus transmission within an area (*23*). Last, our before-and-after comparisons may be confounded with seasonal and longer term trends in poliovirus transmission. However, in Nigeria and Pakistan, the median months for IPV+tOPV campaigns were May and June, respectively, compared with June and July, respectively, for tOPV campaigns; the similarity of this timing suggests that the comparison of these campaigns was not confounded by seasonal trends. In addition, the absence of any effect of bOPV against cVDPV2 in Nigeria, as was expected, suggests that any confounding, if present, was minimal.

Our analysis offers support for the use of IPV in mass vaccination campaigns to stop poliovirus transmission, provided good coverage can be achieved. This use of IPV in campaigns should be pursued while maintaining sufficient supply of IPV for routine immunization in high-risk OPV-using countries (*24*). Any vaccine shortage could be compensated for by the use of fractional dose IPV, provided there is sufficient evidence of its efficacy in boosting immunity. The greater effect in Nigeria of IPV+tOPV campaigns compared with tOPV-only campaigns suggest that IPV-only campaigns may also offer substantial benefit. However, campaigns with IPV alone have not been implemented. Evaluation of the potential benefits of IPV-only campaigns in the context of waning intestinal immunity, a growing cohort of children who have not received OPV2, and global containment of OPV2 after withdrawal of the vaccine serotype should be a priority. Results of continuing programmatic experience will also inform efforts to improve the levels of coverage and the benefits of including children 3–4 years of age in IPV campaigns. Regardless, it is now clear that IPV will play a major role in securing the global eradication of poliomyelitis and in maintaining a polio-free world.

Technical Appendix Details of the estimates of the effect of campaigns with different vaccine type(s) on poliovirus detection in Nigeria and Pakistan and sensitivity analyses of these estimates to the choice of time period.
