# Supplementary material for: Estimated Effect of Inactivated Poliovirus Vaccine Campaigns, Nigeria and Pakistan, January 2014–April 2016
Source: Emerg Infect Dis. 2017 Feb;23(2):258–63. doi: 10.3201/eid2302.161210 (PMC5324799; doi:10.3201/eid2302.161210)
Supplement: Technical Appendix — Details of the estimates of the effect of campaigns with different vaccine type(s) on poliovirus detection in Nigeria and Pakistan and sensitivity analyses of these estimates to the choice of time period. [file 16-1210-Techapp-s1.pdf]

Article DOI: http://dx.doi.org/10.3201/eid2302.161210

Estimated Effect of Inactivated Poliovirus Vaccine Campaigns, Nigeria and Pakistan, January 2014–April 2016

Technical Appendix

Technical Appendix Table 1. Incidence of poliomyelitis associated with cVDPV2 and prevalence of this virus in environmental samples in Nigeria in the 90-d period before and after campaigns with different vaccine types*

Vaccine type(s) and state	No. campaigns†	Poliomyelitis incidence (no. cases/100,000 child-years)‡				Virus prevalence in environment, % (no. positive samples/no. tested)			
		Before campaign	After campaign	IRR (95% CI)§	p value	Before campaign	After campaign	PR (95% CI)§	p value
IPV + tOPV									
All	55	0.82 (12/14.59)	0.14 (2/14.59)	0.17 (0.039–0.783)	0.023	13.2 (7/53)	1.9 (1/53)	0.16 (0.019–1.334)	0.09
Adamawa	0	-	-			-	-		
Borno	27	1.94 (11/5.67)	0.18 (1/5.67)			25.0 (3/12)	0.0 (0/10)		
FCT, Abuja	0	-	-			-	-		
Jigawa	0	-	-			-	-		
Kaduna	2	0.00 (0/1.01)	0.00 (0/1.01)			12.5 (1/8)	0.0 (0/8)		
Kano	8	0.00 (0/4.22)	0.00 (0/4.22)			0.0 (0/13)	0.0 (0/13)		
Katsina	0	-	-			-	-		
Kebbi	0	-	-			-	-		
Lagos	0	-	-			-	-		
Sokoto	3	0.00 (0/0.90)	0.00 (0/0.90)			15.8 (3/20)	0.0 (0/19)		
Yobe	15	0.36 (1/2.79)	0.36 (1/2.79)			-	33.3 (1/3)		
tOPV									
All	2759	0.01 (7/639.85)	0.01 (4/555.62)	0.59 (0.18–1.97)	0.391	4.5 (18/397)	2.5 (9/353)	0.45 (0.21–0.95)	0.036
Adamawa	97	0.00 (0/17.84)	0.00 (0/14.66)			0 (0/20)	0 (0/14)		
Borno	90	0.00 (0/16.60)	0.00 (0/15.42)			0 (0/33)	2.4 (1/42)		
FCT, Abuja	24	0.00 (0/6.73)	0.16 (1/6.29)			0 (0/20)	0 (0/19)		
Jigawa	108	0.09 (2/21.96)	0.00 (0/18.83)			0 (0/27)	4 (1/25)		
Kaduna	96	0.00 (0/29.77)	0.00 (0/26.56)			18.2 (6/33)	9.1 (3/33)		
Kano	176	0.09 (4/45.29)	0.05 (2/39.97)			21.9 (7/32)	7.1 (2/28)		
Katsina	136	0.03 (1/29.61)	0.00 (0/25.47)			0 (0/39)	0 (0/25)		
Kebbi	84	0.00 (0/16.87)	0.00 (0/14.42)			0 (0/39)	0 (0/29)		
Lagos	54	0.00 (0/38.46)	0.00 (0/32.64)			0 (0/53)	0 (0/42)		
Sokoto	102	0.00 (0/18.88)	0.00 (0/16.61)			6.4 (5/78)	2.7 (2/73)		
Yobe	56	0.00 (0/8.66)	0.12 (1/8.16)			0 (0/23)	0 (0/23)		
bOPV									
All	838	0.04 (3/84.82)	0.04 (3/71.09)	1.14 (0.23–5.62)	0.868	12.5 (9/72)	25.4 (16/63)	1.76 (0.82–3.78)	0.145

Vaccine type(s) and state	No. campaigns†	Poliomyelitis incidence (no. cases/100,000 child-years)‡				Virus prevalence in environment, % (no. positive samples/no. tested)			
		Before campaign	After campaign	IRR (95% CI)§	p value	Before campaign	After campaign	PR (95% CI)§	p value
Adamawa	21	0.00 (0/0.05)	-			0 (0/1)	-		
Borno	51	0.00 (0/4.99)	0.47 (2/4.25)			60 (3/5)	78 (7/9)		
FCT, Abuja	0	-	-			-	-		
Jigawa	79	0.00 (0/7.26)	0.00 (0/6.91)			-	-		
Kaduna	72	0.00 (0/11.36)	0.00 (0/8.94)			20 (3/15)	0.0 (0/8)		
Kano	133	0.20 (3/14.93)	0.07 (1/14.37)			18 (2/11)	30 (3/10)		
Katsina	107	0.00 (0/10.97)	0.00 (0/7.73)			13 (1/8)	14 (1/7)		
Kebbi	66	0.00 (0/5.39)	0.00 (0/3.94)			0 (0/13)	0.0 (0/4)		
Lagos	0	-	-			-	-		
Sokoto	61	0.00 (0/4.53)	0.00 (0/5.23)			0 (0/19)	20 (5/25)		
Yobe	49	0.00 (0/3.64)	0.00 (0/3.25)			-	-		

*bOPV, bivalent oral poliovirus vaccine; FCT, Federal Capital Territory; IPV, inactivated poliovirus vaccine; IRR, incidence rate ratio; PR, prevelance ratio; tOPV, trivalent oral poliovirus vaccine; -, indicate no district-campaign observations for that vaccine type and state.
†Campaigns in each district were counted separately.
‡Children 0–14 y of age, corresponding to the age range for acute flaccid paralysis surveillance.
§By mixed-effects regression.

Technical Appendix Table 2. Sensitivity analysis of incidence rate and prevalence ratios estimated from clinical and environmental data from Nigeria to the period of time considered before and after each campaign*

Ratio and vaccine type	Time period				
	30 d	60 d	90 d	120 d	150 d
IRR (95% CI)					
IPV+tOPV	0.85 (0.13–5.64)	0.27 (0.056–1.28)	0.17 (0.039–0.78)	0.16 (0.036–0.72)	0.22 (0.063–0.770)
tOPV	2.18 (0.41–11.6)	1.40 (0.42–4.69)	0.59 (0.18–1.97)	1.11 (0.40–3.11)	0.55 (0.17–1.82)
bOPV	1.86 (0.78–4.47)	4.12 (0.96–17.70)	1.14 (0.23–5.62)	12.3 (0.57–265)	NA
PR (95% CI)					
IPV+tOPV	1.29 (0.13–12.5)	0.38 (0.046–3.19)	0.16 (0.019–1.33)	NA	0.052 (0.006–0.424)
tOPV	0.73 (0.32–1.69)	0.47 (0.22–1.00)	0.45 (0.21–0.95)	NA	0.37 (0.18–0.76)
bOPV	1.36 (0.75–2.46)	1.59 (0.83–3.02)	1.76 (0.82–3.78)	NA	NA

*bOPV, bivalent oral poliovirus vaccine; IPV, inactivated poliovirus vaccine; IRR, incidence rate ratio; NA, regression estimate did not converge; PR, prevalence ratio; tOPV, trivalent oral poliovirus vaccine.

Technical Appendix Table 3. Incidence of poliomyelitis associated with serotype-1 wild-type poliovirus and prevalence of this virus in environmental samples before and after vaccination campaigns with different poliovirus vaccine, Pakistan, January 2014–April 2016*

Vaccine type(s), Pakistan state	No. campaigns†	No. cases/100,000 child-years‡				Environmental prevalence, %, (no. positive samples/total no. tested)			
		Before campaign	After campaign	IRR (95% CI)§	p value	Before campaign	After campaign	PR (95% CI)§	p value
IPV+tOPV									
All	133	0.15 (19/122.65)	0.16 (14/87.44)	1.01 (0.50–2.02)	0.979	23.0 (34/148)	21.2 (22/104)	0.88 (0.56–1.38)	0.574
Balochistan	11	1.44 (10/6.96)	0.48 (3/6.21)			33.3 (12/36)	31.3 (10/32)		
FATA	4	0.48 (1/2.10)	3.07 (6/1.95)			–	–		
Islamabad	0	–	–			–	–		
Khyber Pakhtunkhwa	59	0.15 (7/48.06)	0.20 (5/25.46)			23.5 (8/34)	11.8 (2/17)		
Punjab	8	0.00 (0/22.34)	0.00 (0/22.34)			6.3 (1/16)	21.4 (3/14)		
Sindh	51	0.02 (1/43.20)	0.00 (0/31.48)			21.0 (13/62)	17.1 (7/41)		

		No. cases/100,000 child-years‡				Environmental prevalence, %, (no. positive samples/total no. tested)			
Vaccine type(s), Pakistan state	No. campaigns†	Before campaign	After campaign	IRR (95% CI)§	p value	Before campaign	After campaign	PR (95% CI)§	p value
tOPV									
All	590	0.31 (173/553.00)	0.24 (134/547.16)	0.79 (0.63–0.99)	0.039	23.6 (70/296)	22.8 (73/320)	0.93 (0.71–1.22)	0.586
Balochistan	117	0.28 (7/24.79)	0.41 (10/24.31)			40.0 (6/15)	28.0 (7/25)		
FATA	61	6.69 (115/17.19)	4.42 (69/15.63)			–	–		
Islamabad	10	0.00 (0/7.10)	0.00 (0/7.41)			18.2 (2/11)	0.0 (0/12)		
Khyber Pakhtunkhwa	67	0.58 (32/55.31)	0.54 (36/66.66)			27.6 (8/29)	27.1 (13/48)		
Punjab	141	0.01 (3/330.27)	0.00 (1/314.73)			13.4 (21/157)	15.0 (22/147)		
Sindh	126	0.15 (16/105.38)	0.17 (18/106.04)			39.3 (33/84)	35.2 (31/88)		
bOPV									
All	421	0.07 (12/174.80)	0.08 (7/85.10)	1.02 (0.41–2.58)	0.963	29.3 (17/58)	27.7 (13/47)	0.98 (0.58–1.67)	0.954
Balochistan	71	0.28 (2/7.26)	0.37 (1/2.73)			40.0 (4/10)	20.0 (1/5)		
FATA	21	0.00 (0/0.49)	0.00 (0/0.03)			–	–		
Islamabad	2	0.00 (0/0.02)	-			–	–		
Khyber Pakhtunkhwa	66	0.20 (5/24.39)	0.06 (1/17.27)			16.7 (2/12)	15.4 (2/13)		
Punjab	92	0.01 (1/100.50)	0.02 (1/42.76)			0.0 (0/16)	7.7 (1/13)		
Sindh	125	0.11 (4/36.73)	0.19 (4/21.10)			55.0 (11/20)	56.3 (9/16)		

*Data are for the 90-d period before and after the campaigns. bOPV, bivalent oral poliovirus vaccine; FATA, Federally Administered Tribal Areas; IPV, inactivated poliovirus vaccine; IRR, incidence rate ratio; PR, prevalence ratio; tOPV, trivalent oral poliovirus vaccine; -–, indicate no district-campaign observations for that vaccine type and state.
†Campaigns in each district were counted separately.
‡Children 0–14 y of age, corresponding to the age range for acute flaccid paralysis surveillance.
§By mixed-effects regression.

Technical Appendix Table 4. Sensitivity analysis of incidence rate and prevalence ratios estimated from clinical and environmental data from Pakistan to the period of time considered before and after each poliovirus vaccination campaign*

Ratio and vaccine type	Time period				
	30 d	60 d	90 d	120 d	150 d
IRR (95% CI)					
IPV+tOPV	2.12 (0.64,7.10)	0.82 (0.34,1.97)	1.01 (0.50,2.02)	0.75 (0.39,1.42)	0.56 (0.33,0.95)
tOPV	0.75 (0.52,1.06)	0.88 (0.69,1.14)	0.79 (0.63,0.99)	0.80 (0.64,1.00)	0.67 (0.53,0.86)
bOPV	0.56 (0.38,0.84)	0.91 (0.51,1.63)	1.02 (0.41,2.58)	NA	0.49 (0.039,6.293)
PR (95% CI)					
IPV+tOPV	0.50 (0.21,1.20)	0.75 (0.44,1.28)	0.88 (0.56,1.38)	0.71 (0.46,1.10)	0.85 (0.59,1.23)
tOPV	1.00 (0.67,1.48)	0.94 (0.71,1.26)	0.93 (0.71,1.22)	1.01 (0.78,1.32)	1.00 (0.76,1.30)
bOPV	0.77 (0.55,1.08)	0.93 (0.59,1.48)	0.98 (0.58,1.67)	1.00 (0.41,2.43)	0.61 (0.087,4.332)

*bOPV, bivalent oral poliovirus vaccine; IPV, inactivated poliovirus vaccine; IRR, incidence rate ratio; NA, regression estimate did not converge; PR, prevalence ratio; tOPV, trivalent oral poliovirus vaccine.
